# Impact of epilepsy on the risk of hospital-treated injuries in Finnish children

**DOI:** 10.1016/j.ebr.2023.100587

**Published:** 2023-01-16

**Authors:** Liisi Ripatti, Laura Puustinen, Päivi Rautava, Mari Koivisto, Leena Haataja

**Affiliations:** a Department of Pediatric Surgery, Turku University Hospital and University of Turku, Turku, Finland; b Department of Public Health, University of Turku, Turku, Finland; c Turku Clinical Research Centre, Turku University Hospital, Turku, Finland; d Department of Biostatistics, University of Turku, Turku, Finland; e Children's Hospital, Pediatric Research Centre, Helsinki University Hospital, University of Helsinki, Helsinki, Finland

**Keywords:** Injury, Child, Prevalence, Trauma, Generalized epilepsy, Focal epilepsy

## Abstract

**Objectives:**

To study the effect of epilepsy on the risk of injury in children.

**Methods:**

All first-born singleton children (n = 133055) born in 2001 – 2006 in Finland were included. Data was collected from national registers up to the first hospital-treated injury during the five years following the onset of epilepsy. Four matched controls were chosen for every subject.

**Results:**

Epilepsy had been diagnosed in 0.66 % of children. During follow-up, 12 % of 884 children with epilepsy and 9 % of 3536 controls were hospitalized for injuries (HR 1.387 [95 % CI 1.115 – 1.725]; *p* = 0.0033). Risk for injuries was higher in boys than girls (p = 0.0057). Mean age at the first injury was 6.8 years (SD 3.3, median 7, range 0–13) in subjects and 7.2 years (SD 3.2, median 8, range 1–13) in controls (p = 0.272). The rate of hospitalization did not differ according to the type of epilepsy. The risks of subjects compared to controls were not significantly different concerning the nature of injury or survival.

**Conclusions:**

Children with epilepsy are at increased risk for hospital-treated injuries. The spectrum of injuries and the risk for death due to injuries are not different in children with and without epilepsy.

## Highlights

• Children with epilepsy were at increased risk for hospital-treated injuries.
• The spectrum of injuries was similar in children with and without epilepsy.
• Fractures were the most common type of injury leading to hospital treatment.
• Ulna and radius were the commonest long bones affected by fractures in both groups.
• The risk for death due to injuries were not different in children with and without epilepsy.

## Introduction

The prevalence of epilepsy is estimated to be 4.5 to 5.0 per 1000 European children and adolescents [1]. In a Canadian study of a health region covering 1.4 million people, persons with epilepsy were reported 1.4 times more likely to have a hospital-treated injury than controls adjusted for sex, age, and comorbidities [2]. Among 404 children under 18 years of age, those with epilepsy were injured more often compared to age-matched controls without epilepsy (18 % and 15 %, respectively) [2]. On the other hand, some hospital-based and population studies did not find increased risk in children with epilepsy and typical cognitive development [3], [4].

Previous studies indicate an increase in the risk of fractures, burns, medicinal poisonings, and childhood drowning deaths in children with epilepsy [5], [6], [7], [8]. However, systematic data analyzing all injury types in children with epilepsy and the effect of the type of epilepsy on injury risk are few and controversial. We aimed to study the incidence of hospital-treated injury in children with epilepsy in a nationwide setting, with special reference to the type of epilepsy and the type of injury, in comparison to matched controls. Our hypotheses were that the overall risk is higher in subjects than controls and that epilepsy types with generalized seizures pose a higher risk for injuries than types with focal seizures.

## Subjects and methods

The study data were derived from the following five nationwide registers. The Finnish Central Population Register was used to identify the participants. National data on the mother’s socioeconomic status (SES) at delivery, delivery history, course of pregnancy, and perinatal events up to one week of age were derived from the Finnish Medical Birth Register. Data on congenital malformations were received from the Finnish Register of Congenital Malformations. The Finnish Hospital Discharge Register (FHDR) was used to identify children having epilepsy, other neurodevelopmental and neurodegenerative diseases, injuries, and hospital treatment periods. Data on deaths were collected from the Causes of Death Register. The Medical Birth Register, Hospital Discharge Register, and the Register of Congenital Malformations are maintained by the National Institute for Health and Welfare (THL). The Cause of Death Register and the Finnish Central Population Register are maintained by the Finnish Population Register Centre. The register data were linked using encrypted unique personal identification numbers.

**Fig. 1:**
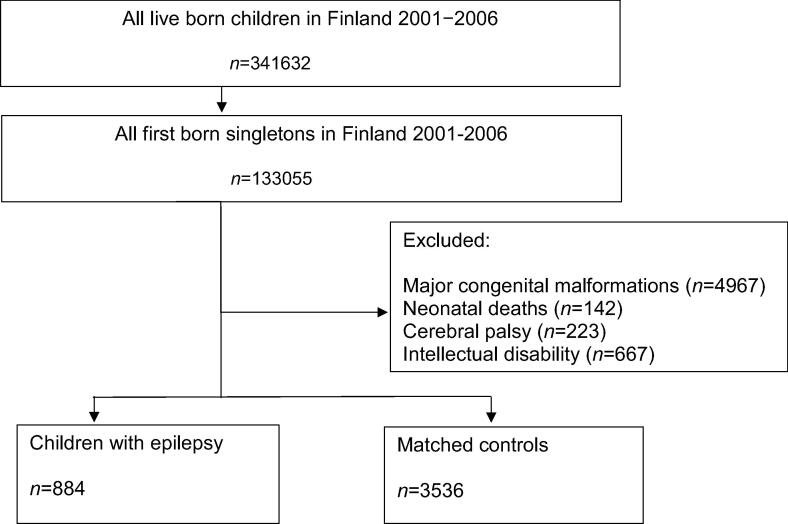
Flow chart of recruitment and data collection.

The source population comprised of all live-born infants born from Jan 1st 2001 to Dec 31st 2006 in Finland (n = 341632). The flow chart of recruitment and data collection is presented in Fig. 1 Of the enrolled subjects, 133,055 were first-born singletons (65,079 females and 67,976 males) who survived the neonatal period and had epilepsy without other initial neurologic impairment including cerebral palsy, intellectual disability, and had no major congenital malformations. We wanted to focus on first-born children without siblings, in order to rule out bias due to large families and busy parents with limited time to guard and protect their children from injuries due to the number of children in the family. In addition, injuries have been shown to be more common in children born as twins or triplets than single-born children [9]. For every subject, four controls healthy for epilepsy, cerebral palsy, and intellectual disability were chosen matched for sex, age (±1 year), and maternal SES.

The data collection period was between January 1st 2001 and December 31st 2014 and five years following the ascertained epilepsy diagnosis. Follow-up of an individual ended at first injury or death. Injuries were classified using the injury mortality diagnosis matrix [10] based on the International Statistical Classification of Diseases and Related Problems 10th Revision (ICD-10). We used the corrected version of the matrix [11]. The matrix includes two axes: one representing the body region and other representing the nature of the injury.

### Definitions

According to the Finnish national Current Care guidelines, all children suspected of having an epileptic seizure are referred to a specialized medical care [12]. Epilepsy is defined according to the classification schemes and guidelines for epidemiological research of the International League Against Epilepsy (Commission 1981, 1989, 1993) criteria [13], [14]. It is mandatory for the hospitals to document the epilepsy diagnosis to the FHDR using ICD-10 codes G40 – G41. Epilepsy type was determined by the first diagnosis of epilepsy recorded to FHDR and classified as follows: 1) focal (G40.00, G40.01, G40.09, G40.10, G40.11, G40.12, G40.19, G40.20, G40.21, G40.22, G40.29), 2) generalized (G40.30, G40.31, G40.33, G40.34, G40.35, G40.36, G40.39, G40.4), and 3) other (G40.80, G40.89, G40.9).

Major congenital anomalies were defined according to the European Surveillance of Congenital Anomalies (EUROCAT) [15]. Maternal SES at delivery was categorized as follows: upper white-collar workers; lower white-collar workers; blue-collar workers; and others (students, homemakers, pensioners). Injuries were defined as any injury requiring hospital outpatient or inpatient care. All co-incident injury diagnoses were taken into account when reporting the distribution of type and nature of injuries in the mortality matrix.

### Statistical analysis

The data were described using percentages and frequencies, or with means, standard deviation (SD), medians and ranges. In addition, incidence rates were calculated. Categorical background variables were compared between children with epilepsy and children without epilepsy using Pearson’s χ^2^ test. Continuous variables were compared using two sample *t*-test. Cox proportional hazard models were used in assessing the risk for any injury in children with epilepsy in comparison to controls, and in children with different types of epilepsy. Epilepsy was first analyzed in a univariate model. The multivariable models included epilepsy, sex, categorized age, and maternal SES. In addition, univariate models including background variables (sex, categorized age and maternal SES) in assessing the risk for any injury were performed for all children and for children with epilepsy. The outcome measure was the first hospital-treated injury. Results are presented with hazard ratios (HR) 95 % confidence intervals (CI), and *p* values. The proportional hazard assumptions were evaluated using log–log survival plot. Univariate associations between the nature of injury and group (epilepsy or control) were analyzed using logistic regression analysis. Two-sided *p*-values less than 0.05 were considered statistically significant. Statistical analyses were carried out using the SAS® system for Windows, version 9.4. (SAS Institute Inc., Cary, NC, USA).

### Ethics

The study protocol was approved by the National Institute for Health and Welfare (THL) (THL/709/5.05.00/2011, THL/470/5.05.00/2013, and THL/595/5.05.00/2019) and Statistics Finland (TK-53–955-13). The register data were linked using encrypted unique personal identification numbers. This was a retrospective register study, and the participants were not contacted. Thus, according to the Finnish law, no informed consent of the participants or Ethics committee approval was required. The legal basis for processing of personal data is public interest and scientific research (EU General Data Protection Regulation 2016/679 (GDPR), Article 6(1)(e) and Article 9(2)(j); Data Protection Act, Sections 4 and 6).

## Results

Epilepsy was diagnosed in 0.66 % (884/133055) of subjects (0.63 % of females and 0.70 % of males) and in none of the 3536 matched controls. The mean age at epilepsy diagnosis was 5.4 years [SD 3.5 years, median 5, range 0 – 13 years], with 230 (26.0 %) aged less than 2 years, 297 (33.6 %) 3 – 5 years, 236 (26.7 %) 7–9 years, and 121 (13.7 %) 6 – 13 years. The mean time from epilepsy diagnosis to the first hospital-treated injury was 4.6 years (SD 1.2, median 5, range 0 – 5 years). In controls, the mean time from the start of follow-up to first hospital-treated injury was 4.7 (SD 0.9, median 5, range 0 – 5). Mean age at first hospital-treated injury was 6.8 years (SD 3.3, median 7, range 0 – 13) in subjects and 7.2 years (SD 3.2, median 8, range 1 – 13) in controls (p = 0.272).

### Background characteristics and the risk of injury

The background characteristics of the study participants are shown in Table 1. In univariate analyses of all study children (including both subjects and controls), male sex (p = 0.0057) and age (categorized) at the start of the follow up were associated with injury risk (p = 0.0002), but not maternal SES (p = 0.3689). Among children with epilepsy, neither sex (p = 0.4393), categorized age at the start of the follow up (p = 0.1678), nor maternal SES (p = 0.4518) were significantly associated with the risk of hospital-treated injury.

**Table 1:** The background characteristics of study participants born in Finland during 2001 to 2006 and the numbers of patients with hospital treated injury during the 5-year follow-up period after epilepsy diagnosis. There were no significant differences in these background characteristics between those with epilepsy and controls (SES p = 0.9997, sex p = 1.000, age p = 0.9976).

Background characteristics	Children with epilepsy		Controls	
	N	N with injury (%)	N	N with injury (%)
**Total**	884	108 (12.2)	3536	320 (9.0)
Mother’s SES at delivery*				
Upper white collar	147	22 (15)	588	61 (10.4)
Lower white collar	289	34 (11.8)	1156	98 (8.5)
Blue collar	133	17 (12.8)	529	48 (9.1)
Other	211	20 (9.5)	836	75 (9.0)
**Sex**				
Male	474	55 (11.6)	1896	202 (10.7)
Female	410	53 (12.9)	1640	118 (7.2)
**Age at onset of follow up**	N		N	
0–2 years	230	28 (12.2)	921	86 (9.3)
3–6 years	297	46 (15.5)	1187	128 (10.8)
7–9 years	236	25 (10.6)	944	84 (8.9)
10–13 years	121	9 (7.4)	484	22 (4.5)

*SES = socioeconomic status, data missing in 12 %.

### Effect of epilepsy on the risk of injury

Out of the children with epilepsy, 12 % (*n =* 108) were injured during the 5-year follow-up after epilepsy diagnosis compared to 9.0 % (*n =* 320) of the controls without epilepsy (in univariate analyses HR 1.387 [95 % CI 1.115 – 1.725], *p =* 0.0033). In multivariable analyses including all participants, the risk of hospital-treated injury was higher in subjects than in controls (HR 1.344 [95 % CI 1.064 – 1.700], p = 0.0133) and in males compared to females (HR 1.309 [95 % CI 1.065 – 1.611], p = 0.0107). Maternal SES was not a significant factor for the risk of injury (p = 0.5044). Children aged 10 – 13 years at the start of the follow up were at significantly lower risk than 0 – 2 -year-olds for injuries (HR 0.567 [95 % CI 0.377 – 0.853], p = 0.0064), while other age groups did not significantly differ from the reference.

The distribution of injuries according to the type of epilepsy is shown in Table 2. There was no significant difference in the rate of hospitalization for injury according to the type of epilepsy (generalized compared to focal and other types) in univariate (p = 0.1377) or multivariable analysis (p = 0.548) including the type of epilepsy (p = 0.1506), sex (p = 0.4428), age at the start of the follow-up, and SES (p = 4481), (p = 0.1805).

**Table 2:** Number of children requiring hospital out- or inpatient treatment for injuries within the 5-year follow-up period according to the type of epilepsy. Results of univariate analysis are presented here.

**Type of epilepsy**	**Total N**	**N with injury (%)**	**HR (95 % CI)**
Generalized	202	22 (10.9)	1.000
Focal	372	39 (10.5)	0.967 (0.574–1.632)
Other	310	47 (15.2)	1.440 (0.868–2.389)

During the follow-up period, 10 patients with epilepsy and 2 controls died (p less than 0.0001). None of the children with epilepsy died of traumatic injury. The causes of deaths of the patients with epilepsy included congenital myopathy (age of death 12 months), metabolic disorder (12 months), brain tumor in three children (4, 11, and 12 months), and pneumonia in five children. All children who died of pneumonia had contributory causes of death: urea cycle metabolism disorder; neuronal ceroid lipofuscinosis; degenerative disease of the nervous system; a sequalae of inflammatory diseases of central nervous system; or concomitant cytomegalovirus and Bordetella pertussis infections.

One of the controls drowned and another extremely prematurely born control with cardiomyopathy and bronchopulmonary dysplasia died of sepsis.

### The nature and the body region of injuries

The most common body regions of injuries leading to hospital care were extremities (in 7.0 % of those with epilepsy and 5.0 % of controls) and head and neck region (3.4 % and 2.7 %, respectively). The body region and the nature of hospital-treated injuries for subjects and controls are shown in Table 3 in Appendix A. The distribution of hospital-treated injuries did not significantly differ by body region between subjects and controls.

Fractures were the most common type of injury leading to hospital treatment in both groups; fractures were the reasons for hospitalization in 3.1 % (27/884) of patients with epilepsy compared to 3.3 % in controls (116/3536) (p = 0.0581, OR 0.612 [95 % CI 0.379 – 1.016]). The distribution of fractures of the long bones did not significantly differ between the study groups. The rate of fractures in the ulna and/or radius was 1.0 % in those with epilepsy and 1.1 % in controls; in the humerus 0.7 % vs 0.8 %; in the tibia and/or fibula 0.6 % vs 0.4 %; and in the femur 0 % vs 0.08 %. Unspecified injuries were more common as a reason for hospitalization in children with epilepsy compared to controls (0.6 %, 5/884 and 0.08 %, 3/3536, respectively, p = 0.0233, OR 5.35 [CI 95 % 1.256 – 22.782]). There were no other significant differences between groups in the nature of injuries leading to hospitalization. Other common reasons for hospital treatment included internal organ injuries (1.6 % and 1.0 %, respectively, p = 2.906, OR 1.270 [95 % CI 0.655 – 2.465]) and open wounds (1.1 % and 1.1 %, respectively, p = 0.4351, OR 0.747 [95 % CI 0.360 – 1.552]). Traumatic brain injuries were the reason for hospitalization in 0.2 % (2/884) of those with epilepsy and none of the controls (p = 0.0595). Concussion led to for hospitalization in 1.2 % (11/884) and 0.9 % (35/3536), respectively (p = 0.9252).

## Discussion

The present nationwide population study shows that children with epilepsy have a significantly higher overall risk for hospital-treated injury than controls (12 % vs 9 %, respectively). Injuries requiring hospital treatment were 1.4 times more likely in children with epilepsy than in controls. No difference in injury rate was found between different epilepsy types. The distribution of injuries by body region and the nature of injury was similar in those with epilepsy and controls. Fracture was the most common type of injury in both groups and extremities, head, and neck the most common location of injury requiring hospital treatment. None of the children with epilepsy died of traumatic injury but one of the controls drowned.

Increased injury risk shown in our study is in line with a Canadian study of one health region covering 1.4 million people also showing a 1.4-fold increase for the whole population and a 1.2-fold increase in the risk of injury for those less than 18 years of age [2]. Several other studies have not separately reported injuries of children and adults [16], [17]*,* even though it is has previously been shown that the types of accidents are age dependent [18]. While we showed that there were few children who suffered a traumatic brain injury, there was a trend towards increased risk in those with epilepsy compared to controls.

Another finding of importance was the lack of any case of drowning or near-drowning in children with epilepsy. Seizure-related drowning has been regarded as a potential risk factor in children with epilepsy [19], and not least in Finland, a country with tens of thousands of lakes with reportedly 10 % of all cases of deaths in persons with childhood-onset epilepsy attributed to drowning [20]. While drowning is a feared and discussed possibility in persons with epilepsy, our results might indicate that the continued guidance of families on the risks of drowning in children with epilepsy has been beneficial. A Danish population-based study showed excess mortality associated with overall accidents among children with epilepsy [21]. Our results were contradictory to this, since there were no deaths directly attributed to injuries in children with epilepsy in our study. Parallel to our results, in recent decades, a worldwide reduction has also been reported in the mortality rate in persons with idiopathic epilepsy [22].

Previous systematically analyzed data on the types of accidents in children with epilepsy are few. Using the comprehensive register data, we showed that even though children with epilepsy needed hospital treatment for injuries more often, the nature and body regions of injuries are distributed very similarly to the controls. This is in line with a Canadian study on children with epilepsy who had a developmental quotient > 70 and had no major motor or sensory impairments [4]. They compared selected injury types in children with epilepsy to controls and showed no significant difference in the number of mouth injuries, lacerations, burns, fractures, head injuries, and bicycle or car accidents. On the contrary, one previous study has shown bicycle accidents have been shown to be increased in children with absence seizures [23].

Children and young adults [7] and adults [24] with epilepsy have previously been shown to be at greater risk for fractures compared to healthy controls. We observed that fractures were the most common injuries requiring hospital treatment among both children with epilepsy and controls. However, our data showed that fractures were actually a more common reason for hospitalization in children without epilepsy than those with epilepsy.

Major strengths of this study are a large cohort size and comprehensive nationwide population-based data. Finnish register data have been shown to be reliable [25]. The Register of Congenital Malformations [26], the Hospital Discharge Register [27], and the Medical Birth Register [28] all have a good coverage. Due to the comprehensive data, we were able to concentrate on the impact of epilepsy on injuries in children by excluding confounders such as intellectual disability and cerebral palsy, that are known to be associated with increased risk for injury [29], [30].

The limitations of the present study include the retrospective time directionality, typical of register studies; limitation to hospital-treated patients; and failure to analyze effects of antiseizure medication on injuries and fractures in the patients. Even though major congenital malformations were excluded, some of the children with epilepsy may have progressive neurological disorders that can cause motor and cognitive impairment and may result in a higher risk of injuries. In addition, while there is no separate epilepsy register in Finland, there were not enough data available to analyze the different seizure types more specifically or to determine whether injuries were sustained during a seizure or as a result of other accidents.

## Conclusions

Children with ascertained epilepsy are at significantly increased overall risk for injuries. While the spectrum of injuries is similar in children with and without epilepsy, children with epilepsy may be more prone to head injuries. Individual preventive measures, such as using helmet and showering in a non-glass cubicle rather than taking a bath, is recommendable especially in difficult-to-treat epilepsies.

## Ethical statement

The study protocol was approved by the National Institute for Health and Welfare (THL) (THL/709/5.05.00/2011, THL/470/5.05.00/2013, and THL/595/5.05.00/2019) and Statistics Finland (TK-53–955-13). The register data were linked using encrypted unique personal identification numbers. This was a retrospective register study, and the participants were not contacted. Thus, according to the Finnish law, no informed consent of the participants or Ethics committee approval was required. The legal basis for processing of personal data is public interest and scientific research (EU General Data Protection Regulation 2016/679 (GDPR), Article 6(1)(e) and Article 9(2)(j); Data Protection Act, Sections 4 and 6).

## CRediT authorship contribution statement

**Liisi Ripatti:** Visualization, Writing – review & editing, Writing – original draft, Methodology, Conceptualization. **Laura Puustinen:** Writing – review & editing, Writing – original draft, Methodology, Conceptualization. **Päivi Rautava:** Supervision, Writing – review & editing, Methodology, Conceptualization. **Mari Koivisto:** Formal analysis, Software. **Leena Haataja:** Supervision, Methodology, Conceptualization, Writing – review & editing.

## Declaration of Competing Interest

The authors declare that they have no known competing financial interests or personal relationships that could have appeared to influence the work reported in this paper.
