# Supplementary material for: Impact of epilepsy on the risk of hospital-treated injuries in Finnish children
Source: Epilepsy Behav Rep. 2023 Jan 16;21:100587. doi: 10.1016/j.ebr.2023.100587 (PMC10015441; doi:10.1016/j.ebr.2023.100587)
Supplement: Supplementary data 1 [file mmc1.doc]

|  | **Body region of injury,** n (% of all) | | | | | | | | | | | | | | |
| --- | --- | --- | --- | --- | --- | --- | --- | --- | --- | --- | --- | --- | --- | --- | --- |
| **Nature of injury** | **Head and neck** | | **Spine and upper back** | | **Torso** | | **Extremities** | | **Unclassifiable by body region** | | **Unspecified** | | **Total** | | |
|  | *Epilepsy* | *Controls* | *Epilepsy* | *Controls* | *Epilepsy* | *Controls* | Epilepsy | Controls | Epilepsy | Controls | Epilepsy | Controls | Epilepsy | Controls | p value |
| **Fracture** | 1 (0.1) | 5 (0.1) | 0 | 1 (0.03) | 0 | 0 | 26 (2.9) | 110 (3.1) | 0 | 0 | 0 | 0 | 27 (3.1) | 116 (3.3) | 0.058 |
| **Dislocation** | 0 | 0 | 0 | 0 | 0 | 0 | 2 (0.2) | 6 (0.2) | 0 | 0 | 0 | 0 | 2 (0.2) | 6 (0.2) | 0.972 |
| **Internal organ injury** | 13 (1.5) | 35 (1.0) | 0 | 0 | 1 (0.1) | 0 | 0 | 0 | 0 | 0 | 0 | 0 (0) | 14 (1.6) | 35 (1.0) | 0.479 |
| **Open wound** | 7 (0.8) | 30 (0.8) | 0 | 0 | 0 | 1 (0.02) | 3 (0.3) | 9 (0.3) | 0 | 0 | 0 | 0 | 10 (1.1) | 40 (1.1) | 0.435 |
| **Amputation** | 0 | 0 | 0 | 0 | 0 | 0 | 0 | 0 | 0 | 0 | 0 | 0 | 0 (0) | 0 | NA |
| **Blood vessel** | 0 | 0 | 0 | 0 | 0 | 0 | 0 | 0 | 0 | 0 | 0 | 0 | 0 (0) | 0 | NA |
| **Superficial and contusion** | 4 (0.5) | 10 (0.3) | 0 | 0 | 1 (0.1) | 8 (0.2) | 11 (1.2) | 24 (0.7) | 0 | 2 (0.06) | 0 | 2 (0.06) | 16 (1.8) | 46 (1.4) | 0.792 |
| **Crushing** | 0 | 0 | 0 | 0 | 0 | 0 (0) | 1 (0.1) | 1 (0.03) | 0 | 0 | 0 | 0 | 1 (0.1) | 1 (0.03) | 0.424 |
| **Burn** | 0 | 0 | 0 | 0 | 1 (0.1) | 4 (0.1) | 1 (0.1) | 0 | 0 | 0 | 0 | 0 | 2 (0.2) | 4 (0.1) | 0.614 |
| **Effect of foreign body entering orifice** | 4 (0.5) | 15 (0.4) | 0 | 0 | 1 (0.1) | 4 (0.1) | 0 | 0 | 0 | 0 | 0 | 0 | 5 (0.6) | 19 (0.5) | 0.670 |
| **Other effect of external causes** | 0 | 0 | 0 | 0 | 0 | 0 | 0 | 0 | 1 (0.1) | 2 (0.06) | 0 | 0 | 1 (0.1) | 2 (0.06) | 0.722 |
| **Poisoning** | 0 | 0 | 0 | 0 | 0 | 0 | 0 | 0 | 1 (0.1) | 0 | 0 | 0 | 1 (0.1) | 0 (0) | NA |
| **Toxic effects** | 0 | 0 | 0 | 0 | 0 | 0 | 0 | 0 | 1 (0.1) | 4 (0.1) | 0 | 0 | 1 (0.1) | 4 (0.1) | 0.816 |
| **Multiple injuries** | 0 | 0 | 0 | 0 | 0 | 0 | 0 | 0 | 0 | 0 | 0 | 0 | 0 (0) | 0 (0) | NA |
| **Other specified injury** | 1 (0.1) | 0 | 1 (0.1) | 2 (0.06) | 0 | 0 | 13 (1.5) | 25 (0.7) | 0 | 0 | 0 | 0 | 15 (1.7) | 27 (0.8) | 0.078 |
| **Unspecified injury** | 0 | 0 | 0 | 0 | 0 | 0 | 5 (0.6) | 3 (0.08) | 0 | 0 | 0 | 0 | 5 (0.6) | 3 (0.08) | **0.023** |
| **Total** | 30 (3.4) | 95 (2.7) | 1 (0.1) | 3 (0.08) | 4 (0.5) | 17 (0.5) | 62 (7.0) | 178 (5.0) | 3 (0.3) | 8 (0.2) | 0 | 2 (0.06) |  | | |
| **p-value** | 0.884 | | 0.980 | | 0.555 | | 0.461 | | 0.827 | | NA | |  |  |  |

Table 3. Numbers of hospital treated injuries in children with epilepsy (n=884) and controls (n=3536) with percentage given in parenthesis. The first hospital treated injuries and co-incident injuries that occurred between 2001 and 2014 and within 5-years after epilepsy diagnosis were classified according to the corrected version of Injury Mortality Matrix (9,10). P-values of comparison between subjects with epilepsy and controls are given. NA=Not applicable, statistical comparison between cases and controls unreliable due to small numbers.
